# Zika Virus: Where Is the Treatment?

**DOI:** 10.1007/s40506-016-0083-7

**Published:** 2016-07-08

**Authors:** Noreen Mumtaz, Jeroen J. A. van Kampen, Chantal B. E. M. Reusken, Charles A. B. Boucher, Marion P. G. Koopmans

**Affiliations:** Department of Viroscience, Unit Clinical Virology, Erasmus MC, Room Na-1018, Wytemaweg 80, 3015 CN Rotterdam, The Netherlands

**Keywords:** Zika virus, Antiviral, Nucleoside analogue

In the twenty-first century, we have seen the (re-)emergence of several RNA viruses causing severe infections in humans, like SARS and MERS coronavirus, and more recently Ebola virus and Zika virus (ZIKV). A problem with (re-)emerging virus infections is the lack of available medical countermeasures, including antiviral treatment. Therefore, the development of broadly acting antiviral compounds, as well as screening of existing drugs for potential repurposing are explored as ways to fast-track the drug development pipeline for emerging viral diseases. Here, we briefly discuss the available information on potential antiviral treatment of ZIKV infection, as well as specific challenges regarding use of antivirals.

Zika virus is a positive-sense single-stranded RNA arbovirus that belongs to the genus *Flavivirus* of the family *Flaviviridae*. Based on nucleotide sequences, ZIKV can be further divided into an African lineage and an Asian lineage. The main vector for ZIKV transmission are *Aedes* mosquitoes. The first isolation of ZIKV was made in 1947 from serum of a febrile rhesus monkey in the Zika forest of Uganda. During the twentieth century, sporadic human cases of ZIKV infection were reported in Africa caused by the African lineage, and in Asia caused by Asian lineage. The epidemiology of ZIKV changed dramatically in the last decade, when major outbreaks of the Asian lineage of ZIKV were reported on the Island of Yap in 2007 and in French Polynesia in 2013. Subsequently, the Asian lineage of ZIKV spread to the Americas in 2015.

The current outbreak of ZIKV shows new modes of transmission with mother-to-fetus transmission and sexual transmission. Furthermore, infectious ZIKV has been detected in breast milk, and there are concerns of ZIKV transmission by blood products obtained from asymptomatic viremic persons or by organ transplantation.

While postnatally acquired ZIKV infections are asymptomatic or associated with mild symptoms, the recent outbreaks brought two severe complications to light: a Guillain-Barre-like syndrome (GBS), associated with paralysis, and microcephaly and other neuro-developmental problems in infants born to women infected with ZIKV during pregnancy. The rate of complications has been estimated to be around 1/4000 cases for the GBS-like syndrome, and may be considerably higher for the teratogenic effects, although much remains unknown about the precise extent of the possible sequelae in fetuses and infants. A significant contributing factor to this knowledge gap is that up to 80 % of Zika virus infections may be asymptomatic, and these ‘silently’ infected women with potentially affected fetuses and babies may go unnoticed.

**Fig. 1:**
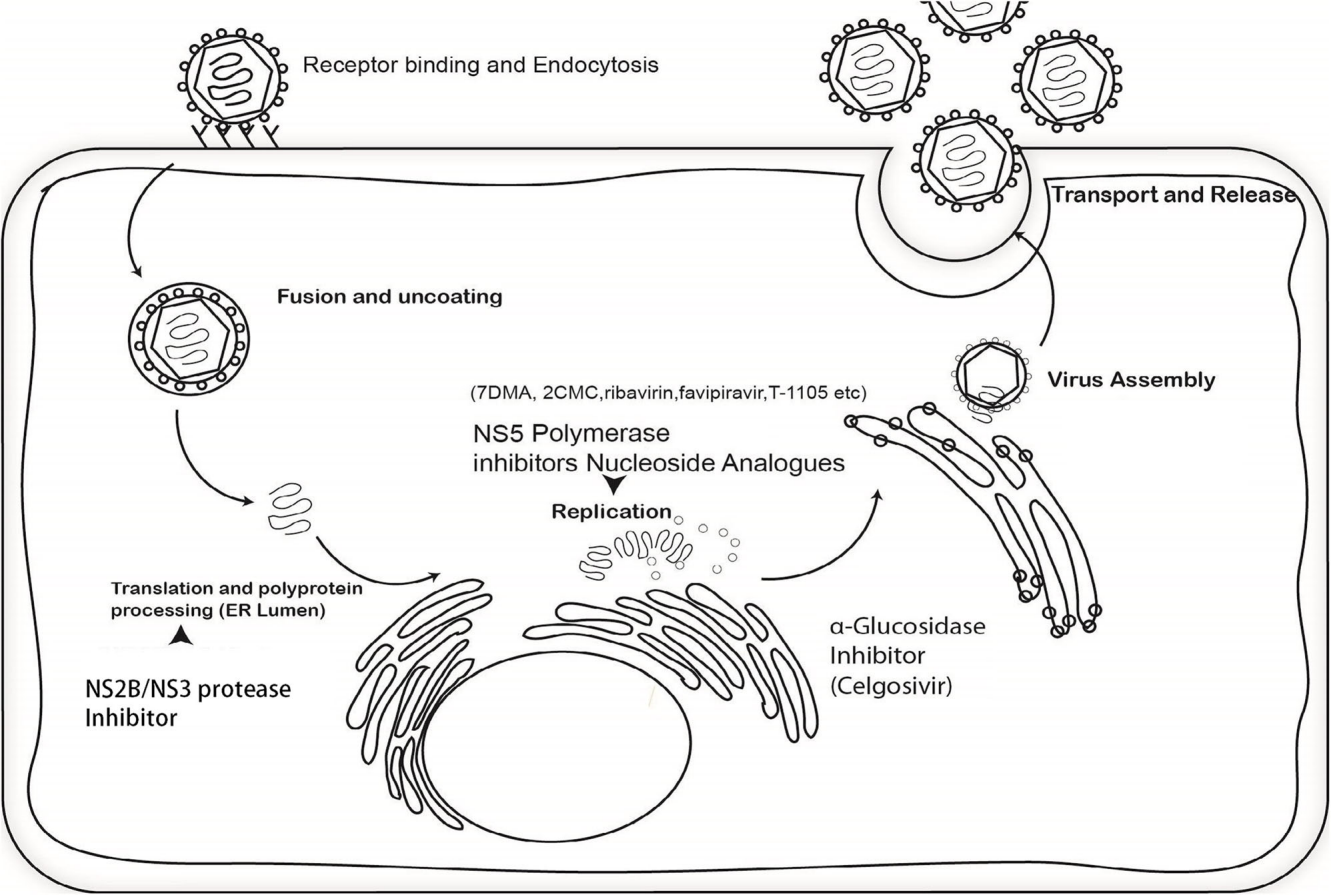
Replication mechanism of ZIKV and potential antiviral targets. Targets shown in the figure are known antiviral targets for other flaviviruses and hepatitis C virus.

There are currently no drugs approved for treatment, but several nucleoside analogue drugs have some antiviral activity in cell culture such as 2′-C-methylated nucleosides like 7-deaza-2′-C-methyladenosine (7DMA) and 2′-C-methylcytidine (2CMC), and ribavirin, favipiravir, and T-1105 (Fig. 1). In addition, 7DMA shows modest anti-ZIKV activity in mice. Ribavirin and favipiravir are already approved for use as antiviral drugs in humans for other indications, but showed less in vitro antiviral effect against ZIKV than 7DMA. 7DMA and 2CMC were initially developed for treatment of hepatitis C virus (HCV) which is distantly related to ZIKV, and studies have shown that these compounds have activity against other flaviviruses much more closely related to ZIKV like dengue virus (DENV). Of interest, other anti-HCV nucleoside analogues like the approved sofosbuvir and mericitabine (in clinical trials) also showed in vitro activity against DENV. GS-5734 and BCX4430 are two very broad spectrum anti-RNA virus nucleoside analogues that are currently in phase 1 and 2 clinical trials. According to the manufacturer, BCX4430 reduced mortality in ZIKV-infected mice, but this data has yet to be published.

The above shows that there is some evidence for the use of broad spectrum antiviral drugs against ZIKV, in particular broad spectrum nucleoside analogues, but the level of evidence thus far is insufficient for licensed clinical applications. Also, a major challenge is how and when such antiviral treatment would be applied, if available. The drug should be active in cells of the nervous system; it should cross the blood-brain-barrier and the placenta, and should be safe for pregnant women, their fetuses, and infants, which are by no means trivial concerns. For example, the unfavorable toxicity profile of ribavirin including teratogenicity limits its use to treat ZIKV infections in pregnant women. In addition, a lesson learned from treating other RNA viruses is that monotherapy can cause the rapid emergence of resistant viruses, stressing the need for development of drugs with different modes of action to reduce the likelihood of selecting out resistant strains.

Given the time needed to develop and approve new antiviral drugs, clinicians are currently left empty handed in terms of targeted antiviral treatment for ZIKV infection. A major lesson from this outbreak and previous (re-)emerging RNA virus outbreaks is that we need safe, broad spectrum antiviral drugs that can be tested rapidly in phase III clinical trials during outbreaks to gain the time needed for the development of other control and prevention measures, particularly vaccines.

